# Path Research on the Value Chain Reconfiguration of Manufacturing Enterprises Under Digital Transformation – A Case Study of B Company

**DOI:** 10.3389/fpsyg.2022.887391

**Published:** 2022-05-17

**Authors:** Yuanqin Li, Hao Ding, Ting Li

**Affiliations:** School of Management, Shanghai University, Shanghai, China

**Keywords:** value chain, reconfiguration path, digital transformation, manufacturing enterprises, case study

## Abstract

Emerging new digital technologies speed up the pace of the layout of the digital economy in various countries. As the main body of China’s real economy, the manufacturing industry does not account for a high proportion of the digital economy. Hence, the digital transformation of the manufacturing industry has become a hot topic. From the view of value chain reconfiguration, we selected B company as the case and introduced data from the closed loop of collection, transmission, storage, processing, and feedback into the analysis of the value chain. This study deeply studies the path of value chain reconfiguration of B company in the context of digital transformation. It finds that, under the impetus of digital transformation, B company has formed a new value chain form which is connected by data nodes in the value chain link and completed the reconfiguration of value chain through value chain boost, value chain integration, and value chain bond. On this basis, the study puts forward countermeasures and suggestions for the next phase of B company’s digital transformation and the digital transformation of the manufacturing industry.

## Introduction

Emerging technologies such as Big Data, Internet of Things, Cloud Computing, and Artificial Intelligence have accelerated human society into an era of the digital economy. Digital transformation is an innovative process in which companies apply digital technologies to adapt to the highly changing digital environment by reshaping corporate vision, strategy, organizational structure, processes, capabilities, and culture (Gölzer and Fritzsche, 2017; Pagani and Pardo, 2017; Chanias et al., 2019; Gurbaxani and Dunkle, 2019; Hess et al., 2020; Verhoef et al., 2021). From macroscopic aspects, the digital economy has become a new engine for global economic development. From micro aspects, companies are faced with challenges and opportunities of digital transformation. The manufacturing industry is the main body of China’s real economy and the key engine to promote long-term stable economic growth. It is of great significance to sustained economic prosperity and social stability. Digital transformation is a process for traditional manufacturing enterprises to break through themselves and pursue innovation (Libert et al., 2016). It has become an important weapon for manufacturing enterprises to support strategic transformation and lead business reform.

Countries around the world have recognized the positive role of digital transformation in manufacturing development and formulated corresponding national strategies, such as “Industry 4.0” in Germany and “Industrial Internet” in the United States (US). The manufacturing industry is a business card of Germany, and the advanced Internet technology and the integration of manufacturing is a feature in the development of the digital economy in Germany. As the pioneer of industrial digitalization Germany first proposed the concept of “Industry 4.0,” leading the world in the field of digital industry. For the United States, “Industrial Internet” mainly contains three elements: intelligent machines, advanced analytics, and workers. It is an open and global network that connects people, data, and machines, aiming to restructure the global industry and stimulate productivity.

China is no exception. In addition to the early policies related to the integration of informatization and industrialization, corresponding development strategies have been intensively introduced, such as “Made in China 2025,” since the 18th National Congress of the Communist Party of China. “Made in China 2025” focuses on digital, networked, and intelligent manufacturing, which embodies the deep integration of information technology and manufacturing technology. It mainly includes eight strategic countermeasures: promoting digital, networked, and intelligent manufacturing; improving product design ability; improving the technological innovation system of manufacturing industry; strengthening the manufacturing foundation; improving product quality; promoting green manufacturing; cultivating enterprise groups and advantageous industries with global competitiveness; and developing modern manufacturing services. According to the data from China Information and Communication Research Institute (2021), the United States is the largest digital economy with 13.6 trillion United States dollars in 2020, followed by China with 5.4 trillion United States dollars, and Germany with 2.54 trillion United States dollars. The scale of China’s digital economy jumped from 2.6 trillion dollars in 2005 to 39.2 trillion dollars in 2020. The proportion of the digital economy in gross domestic product (GDP) also climbed from 14.2% in 2005 to 38.2% in 2020, with an increase of 2.4%. Whether it is “made in China 2025,” “German Industry 4.0,” or “American Industrial Internet,” its strategic core is the national manufacturing transformation and upgrading strategy facing a new era, the fourth industrial revolution and the future. Its goal is to seize the opportunity of intelligent manufacturing and become a powerful intelligent manufacturing country in the new era.

Although China is developing rapidly in the digital economy, it still faces many shortcomings. Firstly, the proportion of the digital economy in GDP is low. The United States digital economy accounted for 66% of GDP, Germany for 66.7%, and China for only 38.2%. There is still a certain gap for China compared with the leading countries. Secondly, the digital level of various industries is uneven. In 2020, 40.7% of China’s service sector was digitalized, while the proportion of manufacturing and agriculture was 21.0% and 8.9%, respectively. The low digitalization of the manufacturing industry and agriculture has become an urgent problem for the balanced development of the digital economy industry in China. Exploring the digital transformation path of manufacturing enterprises has become the key to solving this problem.

Value chain reconfiguration provides a micro theory for understanding the internal reform of corporate digital transformation. Since Porter (1985) put forward the concept of “Value Chain,” the reconfiguration of the value chain has been a hot topic in theoretics and practices. The essence of value chain reconfiguration is that companies flexibly adjust their value activities in the changing process of research and development (R&D), production, and sales to form a new value chain suitable for the market (Tu and Yi, 2018). Unfortunately, the existing research discussed the influencing factors and mechanism of corporate digital transformation from the aspects of digital resources, dynamic capability, and strategic choice (Kohli and Melville, 2019; Warner and Wäger, 2019). The path of value chain reconfiguration, however, is unknown. It is also worth noting that the prior literature on value chain reconfiguration is based on the traditional physical value chain, ignoring the significance of the virtual value chain. A virtual value chain is composed of “information flow” which is the information expression of the physical value chain. Digital transformation emphasizes the factors of data production. Farboodi and Veldkamp (2020) constructed an economic growth model containing data, explained the mechanism of data promoting economic growth, and provided evidence to reduce production uncertainty. The research on the whole data lifecycle is an important breakthrough in deepening the theory of value chain reconfiguration.

Based on the theory of value chain reconfiguration, we analyzed the morphological changes of corporate internal value chain in the process of digital transformation, studied the path of corporate value chain reconfiguration, and emphasized the action mechanism of the whole lifecycle of data elements from collection to feedback. Taking company B of China as a case, we found that manufacturing enterprises use data elements to realize digital transformation through the value chain reconfiguration path of value chain boost, value chain integration, and value chain bond. This study enriched the theory for the digital transformation of the traditional manufacturing industry and provided a reference for other countries, especially developing countries, to cope with a new round of scientific and technological revolution and realize industrial upgrading.

The remainder of this study contains five main sections. First, we sorted out the theories of digital transformation and value chain reconfiguration to construct the theoretical basis of this study and established the connection between the contributions and the theoretical frame of this study. We then introduced research methods, case selection, data collection, data analysis, and other research design contents. Further, we described the process of value chain reconfiguration in three phases of B company. In the penultimate section, we analyzed the influence mechanism of digital transformation on the value chain reconfiguration of B company. Finally, we emphasized the theoretical contributions and put forward the shortcomings and prospects of the study.

## Literature Review

### Digital Transformation

Digital transformation drives the company to use new generation information technologies, such as Big Data, Internet of Things, and Artificial Intelligence, to build a closed loop of data collection, transmission, storage, processing, and feedback, and break through data barriers between different organizations or industries so as to improve the overall operational efficiency of the industry and establish a new digital economy system (Workshop of the Development Research Center of the State Council, 2018). With the development and penetration of digital technology (Matt et al., 2015; Li et al., 2016; Hess et al., 2020), the intensification of competitive environment (Mithas et al., 2013; Kohli and Melville, 2019), and the requirements of personalized user needs (Abrell et al., 2016), digital transformation has become a necessary process for companies, especially manufacturing, to obtain the sustainable competitive advantage (Neumeier et al., 2017).

Digital transformation is a complex and indispensable process of digitization, digitalization, and digital transformation (Verhoef et al., 2021). In addition, the first two elementary phases are needed for the advanced phase of digital transformation (Loebbecke and Picot, 2015; Matt et al., 2015; Parviainen et al., 2017).

The first phase is digitization. The information and communication technology emphasized is that IT technology originated in the 1960s and 1970s (Lu and Wang, 2021). Digitization is mainly to convert analog information into digital information (Yoo et al., 2010; Dougherty and Dunne, 2012; Loebbecke and Picot, 2015; Verhoef et al., 2021), obtain and transmit information accurately and timely, and improve the efficiency of decision-making (Han et al., 2014) and resource allocation (Lai et al., 2010; Vendrell-Herrero et al., 2017). In the digitization phase, the information systems are wholly promoted (Qian and He, 2021), however, the value creation activities are not changed (Verhoef et al., 2021).

The second phase is digitalization, which is not only an information technology means but also a strategic framework to enhance competitiveness (Bharadwaj et al., 2013), is a process to improve operation flows (Van Doorn et al., 2010; Ramaswamy and Ozcan, 2016) and business models (Li et al., 2016; Verhoef et al., 2021). Digitalization eliminates information islands and uses data-driven decision-making and prediction (Qian and He, 2021) in order to save costs and attach importance to customer experience (Pagani and Pardo, 2017).

The third phase is digital transformation. By changing the business scopes (Hess et al., 2020) and value creation processes (Gölzer and Fritzsche, 2017; Verhoef et al., 2021), introducing new business models (Zott and Amit, 2008; Iansiti and Lakhani, 2014; Karimi and Walter, 2015), and influencing the collaborative ways and corporate culture (Warner and Wäger, 2019), the companies achieve the competitive advantages (Casadesus-Masanell and Ricart, 2010; Liu et al., 2011). Digital transformation also makes innovativeness (Svahn et al., 2017), financial performance (Karimi and Walter, 2015), firm growth (Tumbas et al., 2015), and reputation (Yang et al., 2012; Kane, 2016).

There is no denying the fact that digital technologies are needed for digital transformation, however, digital technologies alone provide little value (Kane, 2014). It is their use within the generation of data in a specific context that enables a company to uncover new ways to create value. The manufacturing enterprises are now eager to integrate services and software into core products and turn products into channels for generating, collecting, and exchanging valuable data, exploiting the potential of data for their own benefits (Porter and Heppelmann, 2014). Although most of the literature on digital transformation for manufacturing subscribes the current policy tools (Na and Li, 2021) and application scenarios (Wu and Song, 2020), the transition path (Chi et al., 2021), the microcosmic mechanism, impact of innovation decision (Chi et al., 2020; Zhao and Yang, 2020), and the data value in the digital transformation has not been paid attention to.

### Value Chain and Value Chain Reconfiguration

The value chain analysis is widely used in corporate strategy. Porter (1985) put forward the concept of “Value Chain” and believed that the series of activities carried out by a company in design, production, sales, and other support activities can be demonstrated by the value chain, through which the company completes value creation. Based on this, Shank and Govindarajan (1992) further expanded the scope of the corporate value chain, believing that the value chain of all the companies includes the whole production process from purchasing raw materials from the original suppliers to sending the final products to users. Prior literature analyzed the value chain of manufacturing in optimal control of cost structure (Zhang, 2017), working capital management (Xiao and Jie, 2018), spatial organization mode (Lin et al., 2018), and decision-making model (Hu, 2018).

The value chain is not invariable. A company can fit the changing market environment only if it dynamically adapts to the value chain. Porter believed that the main source of corporate competitive advantage is value chain reconfiguration, which completes whole activities of value creation more effectively. Fine et al. (2002) also described that the real core competence of a company lies in the level of designing and redesigning its value chain. Value chain reconfiguration is an aggregation of original value chain decomposition and new value chain integration (Vogt et al., 2005; Xu and Wang, 2015). The company pays more attention to specialization from the previous comprehensive and balanced development and uses its own comparative advantage to transform into a competitive advantage, so as to reconfigure the value chain (Li and Wang, 2001; Xu and Wang, 2015). Openness, dynamics, and circulation are the three internal characteristics of value chain reconfiguration system (Tu and Yi, 2018).

Recent literature has deeply studied the ways of value chain reconfiguration. They are summarized as counter-flow, multi-dimensionalization, insertion, and removal of a value chain (Wirtz, 2001; Lee, 2005; Hacklin, 2007; Mathur, 2010). Christensen (2006) discussed that value chain reconfiguration itself is a form of value chain innovation, which includes two dimensions: progressive reconfiguration and destructive reconfiguration. Wang and Yang (2015) suggested that in order to adapt to the new development pattern, value chain reconfiguration is needed to achieve longer-term development. Zhang et al. (2021) combined with case studies and found that dynamic capabilities act in turn on the progressive reconfiguration and destructive reconfiguration of the value chain. Progressive reconfiguration mainly focuses on the optimization process of internal value creation nodes, and destructive reconfiguration is committed to changing the link mode with external companies. Wang et al. (2019) claimed that the value chain reconfiguration path under the perspective of creating shared value (CSV) starts from the integration of the internal corporate value chain through the connection between the internal corporate value chain and the global value chain and ends at the aggregation of industrial value chains in different dimensions. Zhang and Yang (2019) researched the garment foreign trade companies under the background of cross-border E-commerce, finding that the value chain reconfiguration path is a coupling process of chain reconfiguration for the internal value chain, coordination for the industrial value chain, and upgrading for the global value chain. Wu and Liu (2021) believed that digital technology promotes the continuous evolution of resource endowment, value proposition, value chain reconfiguration mode, and reconfiguration strategy of publishing companies through three paradigms, organizational empowerment, knowledge empowerment, and network ecological empowerment, to promote the innovative, scalable, and leapfrog reconfiguration of corporate value chain, respectively. Integration through alliances and integration through takeovers and stakeholdings are two methods of integrating new value-added phases (Wirtz, 2001).

In fact, the value creation activities of companies exist in two spaces. One is the material space composed of “physical resource flow,” which is called the physical value chain, and the other is the virtual space composed of “information flow,” which is called virtual value chain. The value chain mentioned above refers more to the physical value chain to some extent. Rayport and Sviokla (1995) put forward the general concept of “virtual value chain” for the first time, believing that “virtual value chain” reflects the physical value chain in the form of information and is the information expression of the physical value chain. The physical value chain and virtual value chain are two parallel value chains of companies. The virtual value chain is based on the physical value chain and is a tool to help to manage the physical value chain. It can create added value and provide strategic guidance for the physical value chain. Traditional value chain management focuses on the value creation activities of “physical resource flow.” Although it also includes the use of “information flow,” it only uses “information flow” to maintain and improve the physical value chain, so it is regarded as an auxiliary tool for value creation. However, in the context of the digital transformation of the manufacturing industry, companies use “information flow” more actively through value mapping, value remodeling, value sharing, and value promotion to restructure and allocate value-added information to realize value creation (Feng, 2020).

These theoretical underpinnings represent the starting point for our investigation on the path of manufacturing value chain reconfiguration under the background of digital transformation. Different from the value chain transformation mode of service industries, such as the media and communication industry or telecommunications industry, driven by industry 4.0, the traditional manufacturing industry is more motivated to urgently complete the transformation and upgrading to realize value-added with the help of digital technology. We believe that digital transformation brings complex changes to a company, involving operating activities, business model, organizational structure, etc. They are not independent and interact and promote each other. So, how does the manufacturing industry realize the digital transformation? Value chain reconfiguration provides a micro theory to answer this question. The internal value chain pays more attention to the analysis of various primary and support activities, which are conducive to opening the “black box” of corporate management. Furthermore, the impact of digital transformation on the corporate value chain not only includes the traditional physical value chain but also emphasizes the role of data in the virtual value chain. As a new economic element, data comes from and acts on the value chain. It is urgent to analyze the effect and mechanism of digital transformation on the manufacturing value chain from the perspective of the whole life cycle of data.

## Research Design

### Research Methods

Based on Eisenhardt’s (1989) and Yin’s (2017) theory, we used the case study method to explore the path of value chain reconfiguration of B company. The main reasons were as follows: first, a case study can effectively analyze the relationship between variables in complex phenomena (Eisenhardt and Graebner, 2007) and clearly solve the “How” problem, which is suitable for the research needs of this study; second, as mentioned in the literature review, the research on the value chain reconfiguration of manufacturing enterprises under digital transformation is still in the exploratory phase, while the case study is suitable to meet the research needs of exploratory research, with the purpose of constructing new theories or compacting specific concepts in existing theories; third, the longitudinal analysis of single case study can carry out a comprehensive analysis of the research object and give a more accurate explanation of complex phenomena.

### Case Selection

We selected B company as the object of the case study. It was based on the following reasons: (1) As one of the earliest steel enterprises established in China, B company has experienced various phases of the development of steel enterprises. Since its listing, it has been at the forefront of the market value in the industry classification of the China Securities Regulatory Commission and maintains the highest credit rating in the global steel industry. In World Steel Dynamics (WSD’s) 2020 ranking of world-class steel enterprises, B company ranked among the top three in terms of steel sales scale, proximity to downstream users, high value-added products, and profitability. In the evaluation of 20 million tons of listed steel enterprises, B company has maintained a leading position in the global steel industry in terms of profit per ton of steel, R&D investment rate, lost time injury frequency rate, and other aspects. Through the introduction and continuous technical transformation, the company maintains the world’s most advanced technology level and has certain representativeness; (2) As a listed state-owned enterprise, B company actively responds to the call of State-owned Assets Supervision and Administration Commission of the State Council (SASAC) to carry out digital transformation and a number of digital-related scientific research projects with relevant ministries and commissions. Moreover, B company’s cold rolling intelligent production workshop is one of the first 20 intelligent factories officially awarded in Shanghai. Particularly, the 1,580 hot rolling intelligent workshop is the first truly unmanned workshop in China’s hot rolling workshop, and the 5G + iron and steel interface intelligent platform launched in 2002 is also the first in China.

### Data Collection

The data of this study included field investigation, in-depth interviews, and public data. Primary data includes field research and in-depth interviews. In this study, we carried out field research on B company many times to get a deep understanding of the corporate structure, business model, and other information. From March 2021 to December 2021, a total of five in-depth interviews were conducted with enterprise managers and technical personnel, and the interviewees were interviewed for more than 360 min. As shown in Table 1, the interview materials are stored in written and recorded forms and converted into written materials for coding. Secondary information includes: (1) Enterprise official website, CNKI, and other channels, including the company’s project, operation mode, system progress, and other information; (2) News reports, including *Shanghai manufacture* and other domestic and foreign media reports; (3) Reports on the digital transformation of B company by WeChat public account. The above data are sorted and classified to form the basic data of the enterprise in this case, as shown in the following table.

**TABLE 1:** Data information and coding.

Collection methods	Data source	Data type	Purpose	Coding
Field research and interviews	5 in-depth interviews were conducted with enterprise managers and technical personnels	Field research and in-depth interviews	Development status of B company, digital technology adopted, changes brought about by digital technology, organizational structure, scientific research projects, etc.	A1-A16
Second-hand information	Official website, CNKI	Authoritative information	Company projects, operation mode, system progress and other information	B1-B11
	*Shanghai manufacture, Internet Weekly* and other domestic and foreign media reports	Media reports	Opinions expressed by company managers in public occasions, strategic layout of the enterprise in recent years, etc.	C1-C8
	WeChat official account	Research reports	Research reports and analytical articles written by relevant institutions for B company Shares	D1-D17

### Data Analysis

A single case study not only needs to “tell a good story,” but also needs to carry out systematic conceptual coding of the research phenomenon and refine universal management theory (Mao, 2020). This study follows the data coding and analysis steps in the case study to construct the theory, and the coding process is divided into four phases: initial coding, focused coding, axial coding, and theory coding (Charmaz, 2006).

Initial Coding: We conducted initial coding on the data collected in the early phase, and the initial coding was required to conduct open theoretical exploration as close as possible to the original data, forming rich topics for the path construction of value chain reconfiguration. After initial coding, complex and disorderly data were gradually clear and easy to refer to and compare, which provided convenience for the subsequent coding and analysis process.

Focused Coding: On the basis of the initial coding, we selected and reduced the interview data and focused on the research questions for independent coding. Specifically, we were divided into two groups, and researchers with experience in qualitative data analysis as the main coders (Nemeth et al., 2001). The coding scheme was initially developed before coding, and key constructs, logical relations, and important processes were summarized and refined. In the process of focusing coding, screening, verification, and adjustment were carried out to form a new coding. Among them, some of the coded constructs were derived from existing literature, and some from our initial summary, which were further refined in subsequent coding until theory saturation was reached. Data were classified with key constructs through focused coding, and relevant theoretical categories were summarized. If there was data omission in focused coding and literature comparison, we would return to the initial coding cycle.

Axial Coding: Based on the focused coding results, we externalized the attributes and dimensions of the theoretical categories, thus connecting the results of the scattered data analyses. Taking the primary category “enhancing core competitiveness” as an example, it was related to the sub-category “optimizing products by combining digital technology with manufacturing” and “transforming process by using digital technology” so as to further refine this category. In addition, we attempted to construct the correlation between categories, excavated the deep connotations of focused coding, and abstracted the key constructs into themes with more theoretical connotations (Strauss and Corbin, 2007), thus fully preparing for theoretical coding. In this process, we jointly checked the coding results and discussed the inconsistent views repeatedly until the consensus was reached to ensure the accuracy and consistency of the coding results.

Theory Coding: we adopted theory coding to construct the theoretical logic among the major genera and formed an interrelated and logically smooth theoretical framework. Through the original data and in coding repeated comparison between the results and literature theory, we formed a preliminary theory frame on the basis of inductive refining and repeated iteration. In addition, by using multiple data sources to test and verify, we gradually realized the data and clearly and reasonably matched the theory of security from data analysis to the theoretical framework of logic. At the same time, we improved the theoretical framework to explain the practical phenomenon. At this phase, through the comparative analysis between the data and theory, the new framework, and the original theory, we found that at present, few scholars focus on introducing data value of the manufacturing enterprise value chain reconfiguration path problem, which clearly put forward that the theoretical framework of this study is comprehensive and innovative and is able to help the development of new theoretical reasoning.

### Theoretical Analysis Framework

The essence of value chain reconfiguration is that enterprises flexibly adjust their value activities to form a new value chain adapted to the market in the ever-changing links of R&D, production, and sales. For manufacturing, in the process of digital transformation, the most direct change brought by the digital technology is the optimization and reform of production process. In addition, it can directly or indirectly produce change to the value chain, of which the most direct change is the value of product itself, and the indirect change is boosting the value of its other links. In addition, in the process of digital transformation, a large amount of data will inevitably be generated. Not only does the data have value itself, but the process from data generation to processing also has a profound impact on the value chain. The mass production and application of data also affects the corporate structure. After accumulating enough data value, departments can more deeply participate in the decision-making of corporate behavior. This will break the original way of communication and connection among departments to promote the realization of value co-creation and benefit sharing and form a substantial community of interests.

**FIGURE 1:**
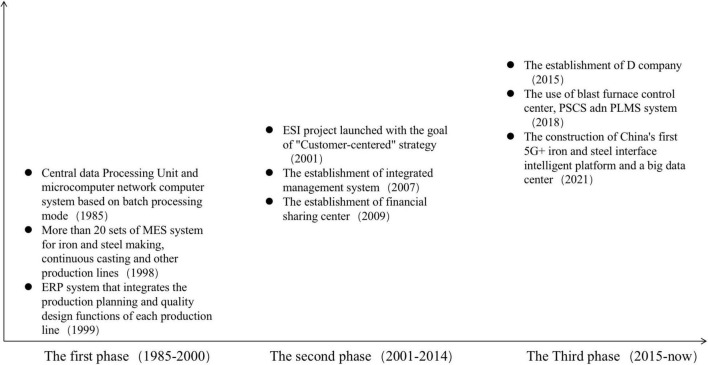
Three phases of digital transformation.

Based on the above analysis, we constructed a theoretical framework for the value chain reconfiguration of B company, as shown in the Figure 1 below. In the first phase of digital transformation, B company focuses the main force of digital transformation on the upgrading of production link to drive the value improvement of the remaining links of the value chain. In the second phase of digital transformation, with the introduction of various digital systems, each link of the value chain completes its own closed loop of data. Through the integration of data value and value chain, the value of each link of the value chain can be improved. In the third phase of digital transformation, under the synergistic effect of each department participating in the value chain, the data nodes of each link in the value chain are connected to promote the synergy between different links in the value chain, thus achieving value-added results.

## Case Description

B company is the world’s leading modern iron and steel joint enterprise. After completing the merger of W steel company, B company had occupied more market share in China. The value chain of an enterprise is composed of the primary activities and support activities that increase the internal and external value of the enterprise. Among them, the primary activities mainly include material creation and sales, transfer of buyers, and after-sales service activities. Support activities are complementary to the primary activities, which are supported by the provision of procurement, technology, human resources, and enterprise-wide functions. For B company, the primary activities of the value chain include internal logistics, external logistics, marketing, and service, while support activities include enterprise infrastructure, human resource management, technology development, and procurement. Each activity includes three parts: direct value creation activity, indirect value creation activity, and quality assurance activity. Primary activities and support activities are distinguished. Based on the Porter Value Chain Analysis framework and combined with the operation mode of B company, the value chain activities of the enterprise are constructed.

We explored the path of value chain reconfiguration through the logic pattern of “motivation, behavior, and results.” Based on the logic, we found the behavior foundation, which was the influence of enterprise digital transformation on the value chain reconfiguration, matched with the motivation, and the result of the value chain reconfiguration could continue to promote the behavior.

### Phase 1

As shown in Table 2, policy driving and coping with the internal demand of B company is the direct reason for choosing digital transformation. On the one hand, *China’s Electronic and Information Industry Development Strategy*, *National Informatization Ninth Five-year Plan and 2010 Vision Goals*, and other policies were conducive to the digital transformation of enterprise. On the other hand, the company made great efforts to introduce foreign technologies, set up as hot rolling regional management system and cold rolling regional management system, and built up “take production as the center, as well as financial as the center” and other early-phase goals of the enterprise, indirectly promoting the process of digital transformation of the enterprise.

**TABLE 2:** Phase 1 value chain reconfiguration.

Logics	Categories	Themes	Examples of coded text units
Motivation	Copying with the internal demand	Achieving the direction of planning	Production-centered
		Research project driving	Conducting various research projects
	Policy driving	Policies are favorable for digital transformation	Emerging a number of digital transformation policies
Behavior	Improving its core competitiveness	Product optimization	More than twenty MES systems are used in the production line
		Revolutionizing the manufacturing process	Strengthening linkages between processes
	Upgrading digital technology	Independent research and development	Central data Processing Unit and microcomputer network computer system based on batch processing mode
		Core business units dominate digitization	The digital work is led by the supervisors of production and business sectors
Results	Value chain boost	Productivity improvement	Improved production efficiency and improved yield
		Other activities increase in value	Enhancing the value of HR management, sales and other activities

With the support of the above motivations, B company improved its core competitiveness through the combination of digital technologies and production activities. The digital trend requires that enterprises can always respond to the external environment pressure, to maintain competitive advantages. The digital work of B company, in surrounding of the production activities, mainly included two ways, namely, the use of digital technologies and the combination of digital technologies and manufacturing to optimize the products. With the combination of information technologies and iron and steel business pilot application as the starting point to seek breakthroughs, B company realized basic automation, the production planning and quality management system, the overall production and marketing management system, the 9,672 system, and other achievements based on the process control system research and development. Through digital technologies, it had continuously improved the technological process and working procedure arrangement, including the merger of working areas and strengthening the connection with working procedures. At the same time, self-owned technologies actively developed on the basis of imported technologies. The digital work was led by the supervisors of production and business sectors in this phase.

“Value chain boost” is the result of value chain reconfiguration in this phase. Through the path of value chain reconfiguration, B company realized a high degree of digitalization of its the production activities, which had boosting effects on other links in the value chain. In this phase, the production and manufacturing process itself achieved a substantial increase in production efficiency and yield, which improved the efficiency of receiving, storing, and distributing production materials to boost the value of logistics activities. The improvement of product quality made the products more competitive and drove the value of sales activities. In addition, the change of personnel brought by the improvement of production technology drove the value of human resource management activities.

### Phase 2

As shown in Table 3, achieving the goals of transformation, upgrading, policy driving, and effective use of data are the motivations for B company to enter the second phase of digital transformation. On the one hand, policies further promoted the digital transformation of the enterprise. After entering the digital era, enterprises generated a large amount of data, which were needed to be stored and processed after the highly digitized production and manufacturing in the first phase. On the other hand, the enterprise management system and mechanism were still not sound. The use of all kinds of systems promoted in reverse the standardization of business processes and improved the efficiency of management. The company put forward the implementation from “taking financial as the center” to “taking the customer as the center,” and the digital transformation helped accomplishing the customer-centered strategic objective.

**TABLE 3:** Phase 2 value chain reconfiguration.

Logics	Categories	Themes	Examples of coded text units
Motivation	Achieving the goal of transformation	Changing in strategic objectives	Customer-centered
		Low management efficiency	Asset operation efficiency and cost control are not ideal
	Policy driving	Favorable policies for digital transformation of enterprises	Emerging a number of digital transformation policies
	Using data effectively	Data redundancy	Generating a lot of incorrect or currently unusable data
Behavior	Setting up a complete digital system	The adoption of new digital systems	The system covers many business areas
		Compatible with existing systems	Fully compatible with existing systems
		Bottom-up information system architecture	Link different hierarchical systems
	Resource integration	Integration of resources in all links	Unified logistics services and other resources
		Integrating information industry resources	C company was established
Results	Value chain integration	The data of each link of the value chain form a closed loop	A closed loop of data collection, transmission, storage, processing and feedback is built
		Each link in the system to support the realization of value-added	Digital systems increase the value of services and the like

Under the support of the above motivations, B company introduced a large number of systems in each link of the value chain in order to achieve the effect of self-closed loop data in each link. First of all, as the heart link of the core business, production activities involved a number of production bases, which actively cooperated in carrying out digital work, adopted a variety of digital systems, and strove to form a closed loop of their own base data. Secondly, departments involved in other links of the value chain gradually began to use digital systems, which covered multiple business fields, such as finance, sales and logistics control, procurement and logistics control, engineering construction, human resources, collaborative office, and so on. The systems also built a closed loop of data collection, transmission, storage, processing, and feedback. Finally, in the process of system construction, the enterprise realized the target that “data never fall to the ground” in different levels of management information systems and formed a bottom-up vertical structure system. In addition, resources were integrated while the system was established. On the one hand, C company was established to collect information industry resources. On the other hand, when each link was added to the system, the company integrated the resources of this link. For example, logistics service resources were managed and controlled by a unified system, realizing unmanned control and automatic planning.

“Value chain integration” is the result of this phase. Each link on the value chain integrated its own digital resources and formed a closed loop of data with the support of the system. In this process, the value of data was integrated into the value chain, and was really gained by the enterprise, thus enhancing the value of each link. For example, the introduction of the logistics system greatly improved logistics efficiency, having an impact on procurement and freight transport, and led to the value-added of procurement activities and service activities. Hence, a new generation of equipment management information system was put into use, which could monitor equipment information in real time and make the basic equipment realize value-added.

### Phase 3

As shown in Table 4, the further demands of improving the efficiency of resource allocation and establishing platforms are the main motivations for B company to enter the third phase of digital transformation. In the second phase, the company integrated each link with its own link, but there was still the problem of low efficiency in the allocation of resources between different links. In addition, with the continuous development of e-commerce, the company also needed to establish platforms to adapt to the development of the digital era and carried out a deeper digital transformation.

**TABLE 4:** Phase 3 value chain reconfiguration.

Logics	Categories	Themes	Examples of coded text units
Motivation	Improving the efficiency of resource allocation	The resource allocation efficiency is low	Internal resource dispersion
	Establishing platforms	Providing personalized service	Establishing D company
	Policy driving	Policies are favorable for digital transformation	Emerging a number of digital transformation policies
Behavior	Strengthening the link between different links	System integration	Marketing, purchasing and finance have become a system
		Department of synergy	The five departments form a control center
	Preparing for data department	Setting up the Department of Big Data and Intelligence	Undertake intelligent manufacturing, big data and other functions
Results	Value chain bond	Different parts of the value chain generate synergy	Production links and infrastructure equipment to produce synergies and so on

With the support of the above motivations, each link of B company began to bond and produce internal integration. In addition, the advantages of each link of the optimized value chain were positively stacked. On the one hand, in the production and manufacturing process of the core business, the world’s first large blast furnace control center independently integrated by B company was officially put into use. The blast furnace control center could realize centralized operation control, production management, and remote technical support for the four blast furnaces in L Base. On the other hand, the operation of the new generation of equipment management information system, the “iEQMS” system, made the connection between the production link and the equipment link, and the new stock marketing center frame based on the double center (business center + data center) framework made the connections among the service activities, sales interaction, and so on. In addition, the company is also preparing to build a professional data processing department in order to realize the optimization and evolution of information architecture through the construction of big data center, break the “information island,” create a digital ecology of application interconnection, and drive the intelligent and digital transformation of the traditional steel manufacturing industry.

“Value chain bond” is the result of this phase. The data nodes of the links on the value chain produced connections, which made all activities on the value chain more organically linked together, and began to generate synergies. For example, the system integration of marketing, procurement, and finance connected the data collection nodes of sales activities and logistics activities, and the data collection and transportation nodes of production activities linked with the data processing nodes of R&D activities and equipment activities.

## Case Analysis

**FIGURE 2:**
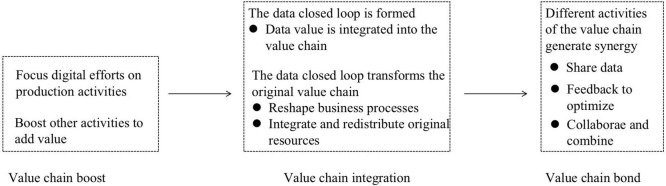
Value chain reconfiguration path.

As mentioned above, in different phases of digital transformation, value chain reconfiguration of B company presents in different ways which can be divided into value chain boost, value chain integration, and value chain bond. After the three phases, the value chain of B company was formed from the traditional value chain to class network value chain, realizing the overall value chain reconfiguration. As shown in Figure 2 below, different value chain reconfiguration methods have different reconfiguration logic in different phases.

### Value Chain Boost

In the early phase of B company’s digital transformation, the digitalization work was concentrated in the production process, and the traditional value chain analysis method could still be used for analysis. Liu et al. (2021) believed that digital management input had obvious differences in its impact on input-output efficiency. Manufacturing execution system (MES)/distributed control system (DCS) projects had more obvious effects in the short term, while enterprise resource planning (ERP) projects and product life-cycle management (PLM) projects promoted corporate input-output efficiency in the long term. MES/DCS projects were all production information management systems for manufacturing workshops. The digital input in production operations could generate value in a short time and had a value-added effect on the enterprise value chain. In the first phase of the digital transformation of B company, the value-added of other activities in the value chain was driven by highly digitalized production activities to form a value chain boost and realized the reconfiguration of the value chain.

It is worth noting that the reconfiguration of value chain was still divided into the reconfiguration of primary activities and the reconfiguration of support activities. In the aspect of primary activities, the improvement of product quality made the sales activities of primary activities add value. The personalization of the product added value to the service activities in the primary activities. In terms of support activities, in the process of digitization of production activities, the updating of equipment added value to the basic equipment in support activities of the value chain. Digitization of manufacturing led to downsize and add value to human resource management activities in support activities.

### Value Chain Integration

The value chain has a profound impact on enterprise management. Every activity in the value chain should create value for enterprises as much as possible and reduce cost. Digitization requires enterprises to design and organize their resource arrangement from a systematic and value-creation-centric perspective (Amit and Han, 2017). Big data formed by digital technology and its disruptive force is accelerating social, economic, political, and systemic change in many areas. Data resources, different from other traditional resources, are difficult to directly produce great value, but the integration of data and the value chain can make it achieve. In each specific link of the value chain, data is difficult to be used as an activity in the value chain due to the different ways of adding value. However, data can assist in completing activities with low cost, high efficiency, and high quality, and monitor various activities with a more refined digital management process.

Data not only improves the value of each link itself, but more importantly improves the efficiency of resource integration in each activity. In the process of establishing digital system, it is necessary to build the underlying logical framework, and to sort out the business process and previous resources of this link. Enterprises use the existing resource combination to supplement and improve the existing capabilities so as to enrich and enhance the capability combination (Sirmon et al., 2011). With a clear understanding of existing resources, these resources can be better utilized and allocated to improve resource utilization. In this process, the redistribution and placement of resources in this link would lead to changes in the value chain, thus leading to the restructuring of the value chain. In addition, the application of each system in the corresponding link of the value chain would force the standardization of the corresponding link process, which was also an important part of the value chain reconfiguration.

At this phase of digital transformation, each link completed the closed loop of data acquisition, transmission, storage, processing, and feedback after the system put into use. For data collection, perfect data collection was conducive to subsequent data processing. For data transmission, fast data transmission helped to improve response speed, facilitate faster analysis of data, and allocation of resources. For data storage, storing data completely was conducive to data precipitation and future big data operation. Lastly, for data processing, efficient data processing helped to improve the utilization rate of data and optimize the overall operation level of the link. Timely data feedback was helpful for decision-making improvement and danger warning in this link.

In a word, the complete system-wide construction enabled all links of the value chain to form their own data closed-loop, which changed the efficiency, cost, and quality of each link and generated value-added of the whole links. At the same time of system construction, business process remodeling and resource integration and redistribution were caused. Under the joint action of the two, value chain reconfiguration was formed through value chain integration.

### Value Chain Bond

Digital transformation has brought about profound changes in organizational structure. Driven by digital technologies, organizations bring subversive changes not only in resource allocation but also in organizational structure through the process of digital transformation. Digital transformation is a rebalancing behavior, in which changes in value creation, and corporate structure, and the use of digital technologies influence each other.

**FIGURE 3:**
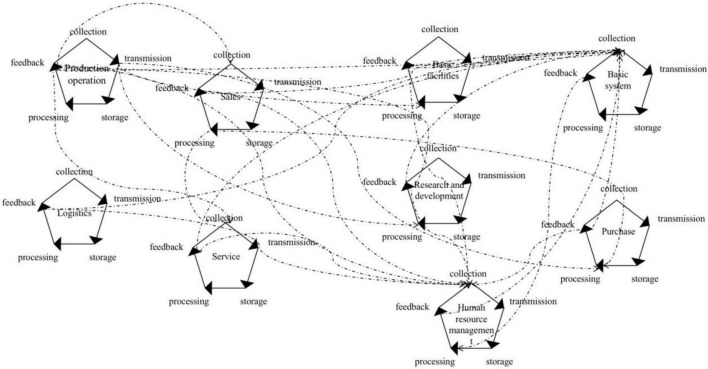
Value chain form.

The concept of “synergetics” was put forward by Haken (1973), a German physicist. He believed that “synergetics” means that each major factor in a large system surmount an individual and produce integration and bond. Later, “collaborative innovation” was proposed by Gloor (2006), who emphasized that collaborative innovation requires network interaction among subjects to exchange ideas, technologies, and information, to finally achieve the goal. Synergy is a long-term, continuous, dynamic and systematic practice process (Xu et al., 2021). Value chain links looped through data nodes and formed a kind of network configuration. As shown in Figure 3 below, it blurred the boundaries between the primary activities and support activities, producing more interaction. The different links of synergy also presented different ways of collaboration. First, the data was shared in a cooperative way. As a manufacturing industry, the most obvious was that the data of production activities was public. The data collected from production activities was used for the production process itself, as well as research and development activities, infrastructure, sales activities, and other links at the same time. Even when providing users with personalized services, such as steel information in real time, data from production activities needed to be provided to service activities for processing. Second, feedback optimized for collaboration. In the feedback phase of each link in the value chain, the data of its own operating system and personnel needed to be fed back to the basic system and human resource management activities for processing so as to optimize the system and human resources, react on other activities of the value chain, and lead to the value-added of other activities. Third, collaboration and mutual integration. After the integration of the company’s marketing, procurement and financial systems, procurement activities in support activities, and sales activities in primary activities integrated from data collection to a large extent. Under the cooperation and mutual integration, some functions of the original value chain link were merged in the perspective of data, which changed the value creation mode of the value chain and completed the reconfiguration of the value chain.

On the whole, B company strengthened the bond with different links of value chain through system integration and departmental coordination, resulting in the synergy. Through data sharing, feedback optimization, cooperation and mutual integration, B company formed the bond of value chain and completed the reconfiguration of the value chain.

### Literature Dialog

#### Manufacturing Value Chain Reconfiguration

Through the boost, integration, and bond of corporate value chain, value chain reconfiguration provides an inexhaustible driving force for the digital transformation of the manufacturing industry. In essence, the process of digital transformation in manufacturing industry is the transformation from “industrialization management mode” to “digital management mode” (Liu et al., 2021). By introducing digital technologies into corporate management structure, it promotes the systematic reconfiguration of information structure, management mode, operation mechanism, and production process compared with the industrialization system and objectively requires the company to break the path dependence under the traditional industrialization management (Huang et al., 2019; Xiao, 2020). Further, changing the original thinking logic of corporate management (Chen et al., 2020), digital manufacturing transformation drives production management to be intelligent, marketing management to be accurate, and resource management to bring subversive innovation of corporate management paradigm and management system (Einav and Levin, 2014; Frynas et al., 2018). We believe that the micro units of these changes are economic activities in companies, that is, the value chain. Digitization and digitalization phases are needed to attain the most pervasive phase of digital transformation (Loebbecke and Picot, 2015; Matt et al., 2015; Parviainen et al., 2017). The representations to three phases of value chain reconfiguration are value chain boost, value chain integration, and value chain bond.

#### The Path of Value Chain Reconfiguration Under Digital Transformation vs. the Path of Value Chain Reconfiguration Under the Situation of Profit Maximization

We found that the similarities and differences between the two approaches were as follows. Firstly, the two approaches have the same starting point, and the value chain reconfiguration in both scenarios starts from optimizing the internal value chain activies. Secondly, the two approaches have different means of reconfiguration. Value chain reconfiguration in the context of profit maximization emphasizes less cross-border and more business subcontracting led by chain splitting (Wang et al., 2019). Value chain reconfiguration, in the context of digital transformation, introduces various digital technologies and means. Thirdly, the path and mode of value chain reconfiguration are different. The value chain reconfiguration under the profit maximization scenario emphasizes the gradual progress of internal value chain, industrial value chain, and global value chain, and the reconfiguration range is from company to industry to region (Zhang et al., 2021). In contrast, digital transformation is more targeted to the internal value chain reconfiguration. Fourthly, the results of the two reconfigurations are different. The value chain reconfiguration under the situation of profit maximization restricts the company to a single industry or a single region, and the company has a clear organizational boundary, while the value chain reconfiguration under the digital transformation builds a new value system.

#### Virtual Value Chain and Physical Value Chain

As an important factor of production, data is born in the internal value chain and acts on it. Virtual value chain provides a theoretical basis for strengthening the importance of data elements in the value chain. However, literature regarded virtual value chain and physical value chain as two chains. In recent years, the coupling relationship between virtual value chain and physical value chain has been proposed (Zhang and Xiao, 2020). Feng (2020) claimed that the value creation activities of virtual value chain under industry 4.0 began with the collection and capture of market consumption information, and then found value-added information through selection, synthesis, and other links. After which, through the reorganization and arrangement of value-added information, realized the value-added of virtual value chain and, finally, practiced the virtual value chain to the physical value chain to create real profits. We believe that the premise of structuring virtual value chain is to generate data. Under the digital transformation, data not only comes from market consumption, but also penetrates into any primary and support activities of physical value chain and acts on every link of value creation. Therefore, virtual value chain is born in the physical value chain. They are neither independent, parallel, nor coupled, but are two sides of the same value chain. With the overall arrangement of digital transformation, each activity of the value chain has formed a closed loop of data. In addition, after the own data accumulation, it has the ability to provide or process data to other activities, so as to generate interaction and finally form a network data chain connected by the data nodes of each link.

## Research Summary

### Research Conclusions

**FIGURE 4:**
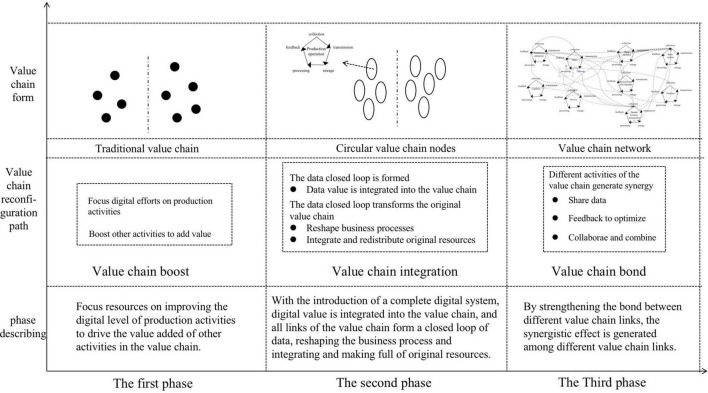
Process model of value chain reconfiguration.

Based on the theory of value chain reconfiguration, this study discovers the different forms of the value chain in the three phases of B company’s digital transformation and summarizes the path of value chain reconfiguration (as shown in Figure 4).

In the first phase, the value of the production link is greatly enhanced through the highly digitalization, to boost the value of other links of the value chain, forming the value chain boost, leading to the reconfiguration of the value chain, and the appreciation of value chain.

In the second phase, the digital value chain is integrated into the value chain by introducing a complete and full-coverage digital system, so that all links of the value chain form a closed loop of data, reshape the business process, integrate and reallocate the original resources, and form the integration of value chain, leading to the reconfiguration of value chain and the addition of value chain value.

In the third phase, through strengthening the bond with different value chain links, the value chain has synergistic effects, including data sharing, feedback optimization, and cooperation and mutual integration, forming value chain bond, leading to value chain reconfiguration and value augmentation.

### Theoretical Contribution

Under the impetus of digital transformation, B company has formed a new value chain form which is connected by data nodes in the value chain and has walked out of its own unique path of value chain reconfiguration. Based on the background of digital transformation and focusing on the internal value chain, this study summarizes the path of value chain reconfiguration of B company and analyzes the impact of digital transformation on the value chain. The theoretical contributions of this study are as follows:

(1) This study expands the theory of value chain reconfiguration. Although a large number of scholars have supplemented and expanded the theory of value chain reconfiguration before, the theory itself should develop dynamically, and the definition of its connotation and dimension needs to be continuously expanded with the changes of time. In this study, the digital transformation phase of manufacturing enterprises is defined by combining data cycle and value chain reconfiguration, and its influence on value chain reconfiguration and reconfiguration path is analyzed, which provides theoretical support for the digital transformation of the traditional manufacturing industry in China.

(2) This study constructs the path model of value chain reconfiguration of manufacturing enterprises, which is embodied in the process of value chain boost, value chain integration, and value chain bond. In the process of value chain analysis, closed loop data is introduced to reveal the process of data integration into value chain, and the reconfiguration of value chain is completed by integrating data value and changing the business process of value chain activities and the way of resource integration. At the same time, this study also analyzes the collaborative ways of value chain links in the digital era, which are mainly divided into data sharing, feedback optimization, cooperation and mutual integration, providing a theoretical explanation for the relationship among different value chain links in the process of value chain reconfiguration.

### Research Deficiencies and Prospects

This study conducts a single case study on B company, but the results have limitations. As a large manufacturing enterprise, B company’s digital transformation has a special impact on the path of value chain reconfiguration based on its single case analysis, which may not be applicable to small and medium-sized manufacturing enterprises. Meanwhile, B company belongs to the steel manufacturing industry. So future study needs to expand relevant research on middle and small-sized enterprises and enterprises in other industries and conduct a more comprehensive analysis on the mechanism of corporate value chain reconfiguration under digital transformation.

## Data Availability Statement

The original contributions presented in the study are included in the article/supplementary material, further inquiries can be directed to the corresponding author/s.

## Author Contributions

YL: responsible for building research ideas and models. HD: responsible for interview design, speech transcription, data coding, case analysis and article writing. TL: responsible for literature review, data collection and coding, model building, and revising the manuscript critically for important intellectual content. All authors contributed to the article and approved the submitted version.

## Conflict of Interest

The authors declare that the research was conducted in the absence of any commercial or financial relationships that could be construed as a potential conflict of interest.

## Publisher’s Note

All claims expressed in this article are solely those of the authors and do not necessarily represent those of their affiliated organizations, or those of the publisher, the editors and the reviewers. Any product that may be evaluated in this article, or claim that may be made by its manufacturer, is not guaranteed or endorsed by the publisher.
